# Subtype-associated differences in HIV-1 reverse transcription affect the viral replication

**DOI:** 10.1186/1742-4690-7-85

**Published:** 2010-10-12

**Authors:** Sergey Iordanskiy, Mackenzie Waltke, Yanjun Feng, Charles Wood

**Affiliations:** 1 Nebraska Center for Virology, School of Biological Sciences, University of Nebraska - Lincoln, 4240 Fair Street, Ken Morrison Life Sciences research Center, East Campus, Lincoln, NE 68583-0900 USA; 2 The George Washington University Medical Center, Department of Microbiology, Immunology and Tropical Medicine, 2300 I Street NW, Ross Hall Rm. 735A, Washington, DC 20037, USA; 3 The D.I. Ivanovsky Institute of Virology, Russian Academy of Medical Sciences, Moscow, Russia

## Abstract

**Background:**

The impact of the products of the *pol *gene, specifically, reverse transcriptase (RT) on HIV-1 replication, evolution, and acquisition of drug resistance has been thoroughly characterized for subtype B. For subtype C, which accounts of almost 60% of HIV cases worldwide, much less is known. It has been reported that subtype C HIV-1 isolates have a lower replication capacity than B; however, the basis of these differences remains unclear.

**Results:**

We analyzed the impact of the *pol *gene products from HIV-1 B and C subtypes on the maturation of HIV virions, accumulation of reverse transcription products, integration of viral DNA, frequency of point mutations in provirus and overall viral replication. Recombinant HIV-1 viruses of B and C subtypes comprising the *pol *fragments encoding protease, integrase and either the whole RT or a chimeric RT from different isolates of the C and B subtypes, were used for infection of cells expressing CXCR4 or CCR5 co-receptors. The viruses carrying different fragments of *pol *from the isolates of B and C subtypes did not reveal differences in Gag and GagPol processing and viral RNA incorporation into the virions. However, the presence of the whole RT from subtype C, or the chimeric RT containing either the polymerase or the connection and RNase H domains from C isolates, caused significantly slower viral replication regardless of B or C viral backbone. Subtype C RT carrying viruses displayed lower levels of accumulation of strong-stop cDNA in permeabilized virions during endogenous reverse transcription, and decreased accumulation of both strong-stop and positive strand reverse transcription products in infected cells and in isolated reverse transcription complexes. This decreased accumulation correlated with lower levels of viral DNA integration in cells infected with viruses carrying the whole RT or RT domains from subtype C isolates. The single viral genome assay analysis did not reveal significant differences in the frequency of point mutations between the RT from B or C subtypes.

**Conclusions:**

These data suggest that the whole RT as well as distinct polymerase and connection-RNase H domains from subtype C HIV-1 confer a lower level of accumulation of reverse transcripts in the virions and reverse transcription complexes as compared to subtype B, resulting in a lower overall level of virus replication.

## Background

Almost 60% of HIV-positive individuals (more than 22 million people) are infected with HIV-1 subtype or clade C. Subtype C is the most rapidly expanding HIV-1 subtype, which predominates in Eastern and Southern Africa and India, and is increasing in frequency in China, Brazil, Uruguay, and nearby countries (reviewed in [1]). In spite of intensive global expansion, no significant differences were observed in the disease progression or pathogenicity of infection in individuals infected by subtype C versus patients infected by other group M subtypes [2]. The epidemic success of subtype C viruses relative to other HIV-1 strains nevertheless suggests that there are factors which may affect the transmission and/or replication of this group of viruses [3]. Although the overall genomic organization is similar among HIV-1 subtypes, sequence diversity between HIV-1 clades may range from 5 to 35% for different genes [4,5]. Indeed, a number of factors related to viral entry and pathogenesis have been indicated as distinct for subtype C HIV-1. They include the predominant use of CCR5 co-receptor by subtype C strains, even in late infection [6,7], and relatively high transmission fitness in dendritic cells, which may increase the frequencies of vaginal shedding and mother-to-child transmission [8,9]. In addition, most subtype C isolates are non-syncytium-inducing which may decrease their cytopathogenicity and hence contribute to the spread of this group of viruses [8,10]. At the viral genomic level, the long terminal repeats have three NF-κB binding sites and a truncation of the Rev protein [11], which may both influence viral replication by enhancing gene expression. There is also a 5-amino-acid insertion in the Vpu polypeptide which may affect the virulence of subtype C viruses through modulation of the Vpu functions, such as CD4 degradation or enhancement of virion release from the cells [12]. Despite these molecular characteristics which may determine enhanced viral replication, the subtype C viruses were found to have lower replication fitness in primary CD4+ T cells and peripheral blood mononuclear cells when compared to all other group M subtypes [8,13,14]. These data suggest there are some viral components of clade C viruses which may decrease the overall replication level or increase the vulnerability of the virus to host restriction factors, but do not alter an enhanced capacity of these viruses to transmit.

The HIV gene *pol *encodes the viral enzymes protease, reverse transcriptase (RT), and integrase and represents the most conserved region of the HIV genome. Nevertheless, differences in the *pol *sequences inherent to certain HIV-1 subtypes have been identified. They include different consensus amino acid (AA) residues in the non-catalytic regions of the protease, RT and integrase. Some of these differences are considered to be subtype-specific signature sequences [15-17], which may potentially affect drug resistance acquisition and probably replicative capacities of the subtypes, as reviewed earlier [18,19]. The protease of subtype C is highly conserved and has differences in the AA sequence when compared to subtypes A, B, and D [3,20]. The subtype C protease has been shown catalytically more efficient than the protease from B subtype, and capable of recognizing more diverse cleavage sites in its substrates [21].

Bioinformatic analysis of the integrase sequences showed that twelve of fourteen subtype C-specific consensus AAs are variable within the subtypes. These consensus residues are located beyond the catalytic triad and functionally important zinc binding motif, LEDGF p75 binding region, and the nuclear localization signal [19,22,23]. Recent investigation of the 3' processing and strand transfer activities of the integrase from subtypes B and C, in the presence and absence of the strand transfer inhibitors, did not reveal any differences in these activities and in susceptibility of these enzymes to the inhibitors [24].

RT is an essential enzyme responsible for HIV replication and determination of the viral variability/polymorphism. The reverse transcription and related events of the virus life cycle have been thoroughly characterized for subtype B viruses (reviewed in [25,26]), while much less information is available for subtype C [5,27,28]. Despite relative conservation of the RT sequence among the HIV-1 subtypes, differences in the effect of RT on virus replication [29], on frequency and location of background polymorphisms [16], and on the development of different resistance patterns in response to treatment with RT inhibitors have been observed between subtypes B and C [15,30,31]. These differences may reflect the functional diversity of RT between subtypes. However, the mechanisms contributing to these differences remain to be determined.

In this study, we hypothesize that RT is the major factor within the *pol*-encoding proteins responsible for subtype-specific differences in the replication of HIV-1. To test this hypothesis, we generated chimeric subtype B and C viruses carrying fragments of the *pol *gene encoding the whole RT, distinct domains of RT, and the protease or integrase sequences from different subtype C and B isolates. In this report we analyzed the basic functions of the Pol-derived proteins in these virus strains, including Gag and GagPol polyprotein processing, accumulation of reverse transcription products in virions and reverse transcription complexes (RTCs), viral DNA integration, the frequency of point mutations in the provirus, and the overall viral replication rates. We did not observe significant differences in the viral protease and integrase activities in viruses carrying the Pol products from B and C subtypes, but found that RT affected replication of the viruses in a subtype-dependent manner. Specifically we showed that viruses carrying RT from subtype C isolates, as well as RT chimeras containing either the subtype C RT polymerase domain or connection and RNase H domains, had decreased levels of viral cDNA accumulation, which correlated with reduced integration and lower levels of viral replication. The frequencies of nucleotide substitutions in the proviral DNA were found to be similar.

## Results

### Characterization of subtype C HIV-1 *pol *genes

**Figure 1:**
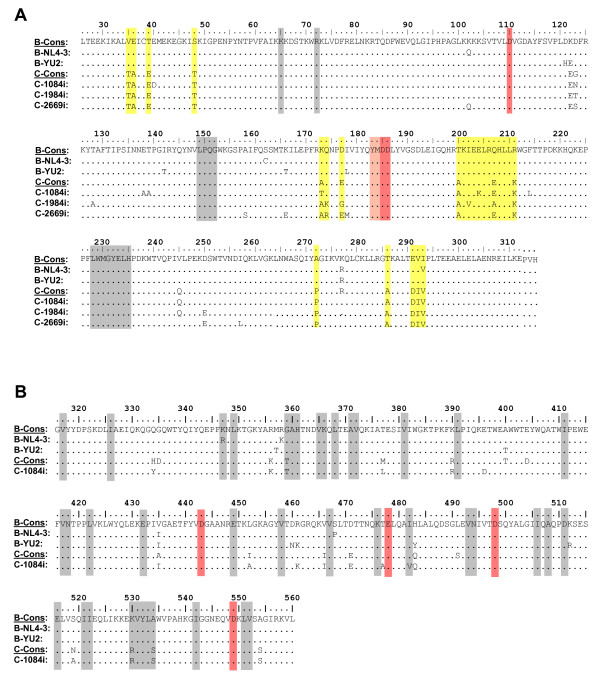
**Comparison of RT sequences of experimental subtype B and C isolates**. All sequences of polymerase domain (AA residues 1-315) (A), connection (AA residues 316-437) and RNase H (AA residues 438-560) domains (B) are aligned with HIV-1 subtype B consensus (upper line). Functionally important RT regions are indicated by the colored boxes: grey - conservative regions: K65, R72 - coordinate triphosphate moiety of dNTPs; LPQG (149-152) - provide proper positioning of incoming dNTPs; LWMGYELH (228-235) - polymerase primer grip; GAH (359-361) - RNase H primer grip; pink - YMDD box: residues 183-186, essential for polymerase activity of RT; orange - catalytic Asp (polymerase and RNase H domains) and Glu (RNase H) residues; yellow - areas of high variability within subtypes. All conservative regions are indicated according to Coté and Roth, 2008 [25].

The *pol *genes of three subtype C HIV-1 isolates were characterized. The viruses were isolated from three perinatally-infected, anti-retroviral naïve Zambian infants. Isolates 1084i and 1984i were obtained from patients with slow disease progression, characterized by a prolonged clinically asymptomatic period (more than four years), whereas isolate 2669i was associated with fast disease progression and a lethal outcome of the infected infant within the first year of life [32]. We also selected two wild-type subtype B strains, NL4-3 (T cell tropic X4 virus) [33] and YU-2 (macrophage tropic R5 isolate) [34] as comparisons. The Pol sequences of these viruses are similar to the subtype B consensus and have only 1.29 (NL4-3) and 1.36% (YU-2) of AA differences from consensus sequence (Los Alamos HIV sequence database http://www.hiv.lanl.gov). The RT fragments within the Pol are relatively more variable and differ from subtype B consensus by 1.6 and 2.3% respectively (Figure 1A and 1B). In contrast, the available subtype C variants are more heterogeneous. The differences of RT AA sequences from subtype C consensus are ranging from 2.4 to 2.9%. Sequences of the RT polymerase domain from analyzed subtype C isolates 2669i, 1984i, and 1084i have from 3.6 to 5.6% diversity among them, whereas the difference between homologous sequences of NL4-3 and YU-2 isolates is 2.6% (Figure 1A). Comparison of the AA clusters in RT, which are distinct between selected isolates and consensus sequences of subtypes B and C indicates that the varying amino acids are not located in the motifs which are critical for the RT enzymatic activity.

### The presence of *gag-pol *or *pol *fragments from HIV-1 subtype C correlates with decreased level of virus replication independently of viral backbone and the cell types

**Figure 2:**
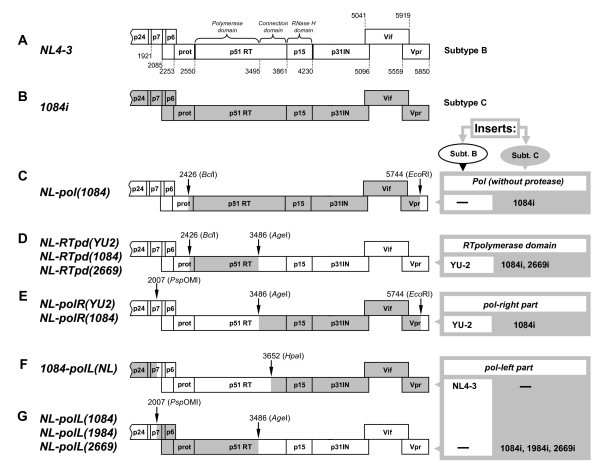
**Generation of recombinant HIV-1 proviral clones comprising fragments of *pol *gene from subtype B and C isolates**. Schematic presentation of the *pol *gene region of subtype B backbone NL4-3 (panel A) and subtype C backbone1084i (B) viruses, recombinant NL-based viruses (C-E and G), and recombinant 1084i-based construct (F). The indicated fragments of the *gag-pol *or *pol *genes from subtype B (isolates NL4-3 and YU-2) or subtype C (isolates 1084i, 1984i and 2669i) proviral DNA were PCR-amplified with primers containing sites of the indicated restriction endonucleases, and inserted into the linearized NL4-3 or HIV1084i proviral vectors to replace the homologous fragments. Selected molecular clones were used for transfection of 293T/17 cells to generate infectious recombinant virus strains.

**Figure 3:**
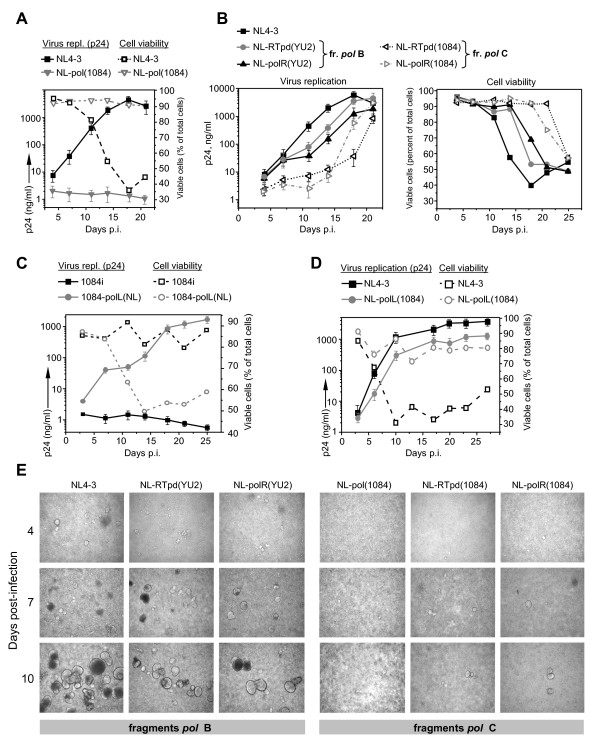
**Presence of the Gag and Pol domains from HIV-1 subtype C correlates with decreased level of virus replication**. **A **- Kinetics of replication (solid lanes) and cytopathicity (dash lanes) of the backbone NL4-3 and chimeric NL-pol(1084) viruses in Sup-T1 cells. The cells (1 × 10^6^) were incubated with virus suspensions (0.01 pg of p24^CA ^per cell) and then cultured in a fresh culture media. Ninety percent of the volume of cell suspensions were harvested every 3 to 4 days, and replaced with uninfected cells. HIV-1 p24^CA ^levels were detected in culture supernatants at the indicated days after infection. Cell viability was measured in cell suspensions using trypan blue staining. Each curve indicating p24^CA ^concentration in the culture media represents the mean data of two independent experiments. Error bars show the standard error. Each curve indicating cell viability represents data of one experiment. **B **- Kinetics of replication (left panel) and cytopathic effect (right panel) of the indicated NL4-3-based viruses in Sup-T1 cells. Infection with virus clones and cultivation of infected cells were performed as described in A. The p24^CA ^curves represent the mean data ± SE from two independent experiments. The curves indicating cell viability represent data from one experiment. **C **- Kinetics of replication and cytopathic effect of the backbone 1084i and chimeric 1084ipolL(NL) viruses in U87.CD4.CCR5 cells. Each viral inoculum (MOI = 0.05) was added to 0.25 × 10^6 ^cells. HIV-1 p24^CA ^concentrations and cell viability were monitored at the indicated days. Each point represents mean p24^CA ^level from two independent experiments. Error bars show the standard error. Each point indicating cell viability represents data of one experiment. **D **- Kinetics of replication (solid lanes) and the cytopathicity (dash lines) of the backbone NL4-3 and chimeric NL-polL(1084) viruses in Sup-T1 cells. Infection with virus clones and cultivation of infected cells were performed as in A. Each curve indicating p24^CA ^concentration represents the mean data ± SE of two independent experiments. Each curve indicating cell viability represents data of one experiment. **E **- Syncytia formation by the Sup-T1 cells infected with the indicated virus strains. Live cells from the experiment described in A and B, maintained in 1 ml of culture medium, were subjected to phase-contrast microscopy on the indicated days after infection. One of ten representative images for each time point is shown.

It has been demonstrated that subtype C viruses do not replicate as well as subtype B and display lower replication fitness in primary CD4+ T cells and peripheral blood mononuclears [8,13,14]. To determine whether the *pol *gene products have a subtype-specific effect on the viral replication, we compared the replication dynamics of a subtype B strain, NL4-3, and a chimeric NL4-3-based virus NL-pol(1084), which carried the 1084i *pol *gene without its protease domain (Figure 2A and 2C), in Sup-T1 cells. Virus replication was monitored by measuring p24^CA^. We found the NL-pol(1084) displayed a much lower level of replication in Sup-T1 cells than the parental NL4-3 virus (Figure 3A, solid lines), as well as less cytopathic effects (Figure 3A, dash lines) and less syncytia in infected SupT1 cell cultures (Figure 3E).

To determine which region of the subtype C *pol *gene affects viral replication, several more chimeric viruses between subtypes B and C were designed, and their replicative capacities and cythopathic effects were tested. We analyzed the replication of two clones NL-RTpd(YU2) and NL-RTpd(1084), which contain sequences encoding the RT polymerase domain only from subtype B isolate YU-2 or subtype C isolate 1084 in the NL4-3 backbone (Figure 2D) (RT domains are indicated according to [35-37]). Another two chimeras carrying the connection domain and RNaseH domain of RT, the integrase, the Vif and the N-terminal portion of Vpr from either the subtype B YU-2, NL-polR(YU2), or from subtype C 1084i isolates, NL-polR(1084), in the NL4-3 backbone were also studied (Figure 2E). All recombinant viruses expressed the backbone NL4-3 *Env *glycoprotein and were tested on SupT1 cells. The presence of either the polymerase domain (pd), or the connection and RNase H domains of RT, integrase and Vif (R) from subtype C 1084i isolate, led to slower viral replication as compared to parental NL4-3 and chimeric viruses carrying homologous fragments from subtype B YU-2 isolate (Figure 3B, left panel). Cytopathic effects of the viruses containing RT fragments from 1084i were proportional to their replicative dynamics, and were reflected in cell killing (Figure 3B, right panel) and formation of syncytia in the infected cell cultures (Figure 3E). To detect whether these differences are subtype-dependent or isolate-dependent, similar chimeric constructs were generated from the other two subtype C isolates: 1984i isolated from a slow disease progressing patient and 2669i from a fast progressor (Figure 2D and 2G). The results were found to be similar to 1084i (data not shown).

Comparison of the replication of the viral strain NL-pol(1084), which carries the subtype C Pol without the protease domain (Figure 3A, grey solid line), with the chimeric viruses NL-RTpd(1084) and NL-polR(1084), containing either the subtype C polymerase domain of RT or the connection and RNase H domains (Figure 3 B, dash lines), shows that after 21 days of infection the first virus displays approximately three logs lower replication level than the other two chimeric viruses. This difference suggests that the N-terminal portion of RT together with the C-terminal Pol domains, the Vif and probably the Vpr proteins may contribute to the lower replication level of the subtype C viruses.

To further determine whether the observed negative effect of the subtype C *pol *gene products on viral replication is independent of the virus backbone, we generated a chimeric virus 1084-polL(NL) containing the protease, RT polymerase domains, and 52 AA residues from the connection domain of subtype B NL4-3 isolate in the 1084i backbone (Figure 2F). In parallel, we generated the NL-based virus carrying a similar fragment of the *pol *gene from subtype C 1084i isolate, encoding the protease and RT polymerase domains without the part of connection domain (Figure 2G). Since 1084i Env is R5 tropic, we then tested the replication dynamics of subtype C-based viruses in U87.CD4.CCR5 cells, whereas the infection with NL4-3 and NL4-3-based chimeric virus was performed in Sup-T1 cells. The chimeric subtype C-based strain carrying the *pol *gene fragment from NL4-3, 1084-polL(NL), demonstrated productive infection with increasing p24^CA ^level and a high cytopathic effect, in contrast to the control wild-type 1084i isolate which resulted in poor viral replication and low cytopathogenicity (Figure 3C). The NL-polL(1084) viral strain containing subtype C *pol *fragment in the subtype B backbone displayed an overall threefold lower p24^CA ^level than the wild-type NL4-3 isolate (Figure 3D). The tested chimeric virus strains were not absolutely identical. The presence of 52 AA sequence of RT connection domain from NL4-3 in subtype C-based virus 1084-pol(NL) could affect the overall level of virus replication. However, the data that both subtype B- and C-based viruses containing the *pol *gene sequences from the subtype C displayed decreased replication level indicate that the subtype C Pol domains to poor viral replication regardless of the subtype B or C viral backbones.

Taken together, our results indicate that the presence of the polymerase domain or the connection and RNase H domains of RT, integrase and Vif from subtype C isolates correlates with slower or low-efficiency replication of chimeric viruses. The presence of both the whole RT and integrase products of *pol *gene from subtype C isolates in subtype B backbone virus strongly decreases the level of viral replication (Figure 3A). This lower replication suggests that the polymerase and C-terminal domains of RT, and likely the integrase protein all contributed to the slower replicative kinetics of the subtype C viruses. On the other hand, the presence of the protease and RT polymerase domain from subtype C isolate 1084i in NL4-3 virus led to a three-fold decrease in viral replication by the 27^th ^day of infection (Figure 3D). Whereas the clone NL-RTpd(1084), containing the same RT sequence without subtype C protease, displayed only slower replication kinetics and reached a similar p24^CA ^level to the NL4-3 backbone by the 21^st ^day of infection (Figure 3B). These data suggest that subtype C protease may also affect the replication of the recombinant viruses.

### The presence of GagPol domains from HIV-1 subtype C does not affect incorporation of viral genomic RNA and maturation of the virions

**Figure 4:**
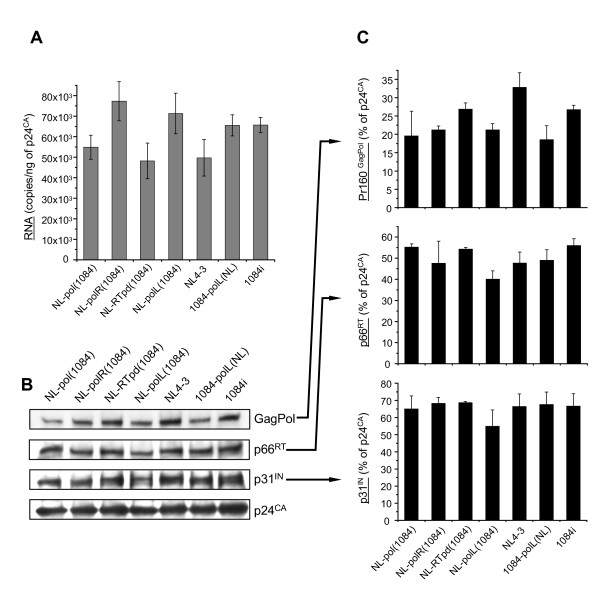
**Recombinant viruses containing the Gag and Pol domains from HIV-1 subtypes B and C do not have differences in RNA incorporation and GagPol processing**. **A **- Quantitation of viral genomic RNA in virus particles. Virus particles were purified from the culture media of 293T/17 cells transfected with molecular clones of viruses at 48 h post-transfection, treated with DNase I RNase free for 2 h and concentrated by centrifugation through 30% sucrose. RNA was isolated from p24^CA^-normalized virus particles, subjected to the reverse transcription with oligo-dT primer and then to quantitative real-time PCR with the primer set specific for positive-strand HIV-1 DNA. The data of analysis of three independent viral preparations were quantified. Each point represents mean RNA copy number ± SD per 1 ng of p24^CA ^in virus sample. **B **- Processing of Pr160^GagPol ^polyprotein-precursor in the virus particles. The virus particles harvested from culture media of transfected 293T/17 cells and purified as in A were analyzed by Western blotting using the antibodies indicated in Materials and Methods. **C **- Quantification of Western blotting results. Western blotting data from two independent experiments were quantified using ImageJ software. Results show mean grey values of the bands ± SE and are presented as percentage of p24^CA ^in each virus sample.

We quantitatively analyzed the incorporation of viral RNA into the virions and processing of GagPol polyprotein-precursor in the virus particles to test the potential effect of the subtype C protease and C-terminal domains of Gag in GagPol chimeras on the precursor protein stability and processing, Gag-RNA binding, and compatibility between the *pol *sequences. Virus particles were harvested from 297T/17 cells transfected with the proviral clones, DNase I-treated, and purified through a 30% sucrose cushion. To quantify viral RNA in the particles, we performed real-time RT-PCR using a primer set recognizing U5-Ψ region of HIV-1 LTR. The results did not reveal any significant differences in viral RNA copy numbers between subtype B and C control viruses and the recombinant viral strains (Figure 4A). Since the protease from B and C subtypes may affect GagPol polyprotein processing (differences are shown in [21]), viral release, dimerization, and total RT count in mature virions differently, we examined the ratio of the products of GagPol processing in the virus particles generated by different viral clones. The Western blot analysis of the purified virus particles was performed with antibodies against p24^CA^, integrase, and RT and with human HIV immunoglobulin which recognizes the Pr160^GagPol ^precursor (Figure 4B). Quantification of Western blotting results relative to p24^CA ^levels for each virus sample did not reveal substantial differences among different viruses (Figure 4C). Collectively, these data demonstrate that the C-terminal domains of Gag and protease from subtype C viruses do not affect incorporation of RNA and the maturation of different recombinant viruses significantly.

### The presence of RT functional domains from HIV-1 subtype C leads to decreased cDNA accumulation in the virions and reverse transcription complexes

**Figure 5:**
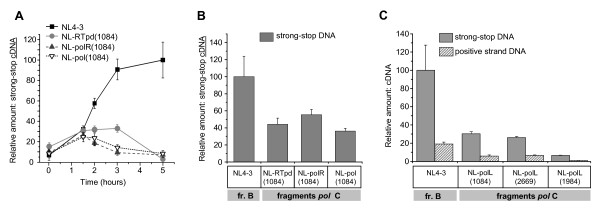
**The presence of RT functional domains from HIV-1 subtype C leads to decreased cDNA accumulation**. **A **- Endogenous reverse transcription (ERT) in permeabilized virions. Purified and p24^CA^-normalized virus particles of either the backbone NL4-3 or NL-based chimeric viruses were subjected to ERT with addition of dNTPs and permeabilizing agent melittin. Samples without dNTPs were used as a control. DNA was harvested after the indicated time of incubation. The relative amounts of negative-strand strong-stop DNA were measured using quantitative real-time PCR. Data from the control samples were subtracted. Levels of cDNA are shown as percentages of the peak accumulation detected in virions of NL4-3 at 5 h after initiation of incubation. Error bars show the standard deviation from three independent viral preparations. **B **- Accumulation of early or strong-stop viral DNA in Sup-T1 cells at 24 h p.i. Untreated or treated with 10 μM nevirapine cells were infected with backbone NL4-3 or the chimeric viruses, containing *pol *fragments from subtype C 1084i isolate using spinoculation. Relative amounts of reverse transcription products were measured using quantitative real-time PCR analysis of DNA from infected cells after incubation with or without 10 μM nevirapine. Data from nevirapine-treated samples were subtracted. Levels of cDNA are shown as percentages of the maximal accumulation detected for cDNA in cells infected with NL4-3 virus strain. Error bars show the standard deviation from three independent viral preparations. **C **- Accumulation of early and late reverse transcription products in Sup-T1 cells infected with recombinant viruses carrying protease and RT polymerase domain from 1084i, 2669i, and 1984i isolates of subtype C at 24 h p.i. The cells were infected with the indicated viruses as described in B. Harvested DNA was measured using quantitative real-time PCR analysis. Levels of cDNA are shown as percentages of the maximal accumulation detected for negative strand strong-stop cDNA in cells infected with NL4-3. Error bars indicate the standard deviation from three independent viral preparations.

To determine why viruses carrying the subtype C RT domains confer lower viral replication than virus strains containing subtype B RT and whether this is due to a difference in reverse transcription, we analyzed the accumulation of reverse transcription products in permeabilized virions, in isolated reverse transcription complexes (RTCs), and in the cytoplasm of cells infected with parental subtype B or C viruses or with chimeric viruses. As reported earlier, reverse transcription of HIV-1 can be initiated within the intact virions [38], and initial steps of endogenous reverse transcription (ERT) taken place before infection can increase HIV-1 replication in some target cells [39]. Therefore, we employed the ERT assay to test the various intact viral particles normalized by p24 ELISA as described earlier [40,41]. The basic level of the early DNA products (negative-strand strong-stop DNA) was found to be very low in all viral particles. In contrast quantitative real-time PCR analysis of the strong-stop cDNA purified from ERT samples after incubation with dNTPs displayed a significant increase in early reverse transcription product only in NL4-3 virions (Figure 5A). Chimeric viruses containing the RT polymerase domain, the connection and RNase H domains, or the whole RT from subtype C 1084i isolate demonstrated an increase of strong-stop cDNA level for the first 1.5 h of incubation, followed by a gradual reduction for the subsequent 3.5 h of incubation.

We analyzed the accumulation of the reverse transcription products in the cytoplasm at 24 h post-infection to identify the effects of the RT from subtypes B and C on reverse transcription in infected cells. To exclude the possibility that differences in viral DNA content in the cytoplasm can be caused by natural ERT and to assess the ratio of DNA synthesized only in the cytoplasm, we synchronously infected Sup-T1 cells by different viruses in the presence or absence of 10 μM non-nucleoside RT inhibitor nevirapine. We then determined the amount of HIV-1 DNA by quantitative real-time PCR. The amount of strong-stop cDNA from the cytoplasm of nevirapine-treated cells due to natural ERT was subtracted so that only DNA synthesized within the infected cells was measured. We found an approximately twofold lower count of the strong-stop DNA in the cells infected with NL-1084 recombinants (Figure 5B). We do not believe that this difference is due to the ability of nevrapine to inhibit subtype B and C RT differently, because it has been shown that *in vitro *10 μM nevirapine inhibited wild-type RTs from both subtype B and C viruses by over 100-fold [28].

Analysis of the cDNA accumulation in Sup-T1 cells infected with recombinant viruses carrying C-terminal Gag products, protease, and RT polymerase domains from different subtype C isolates (1084i, 2669i and 1984i) displayed a significantly decreased level of both early (strong-stop DNA) and late (positive strand DNA) reverse transcription products at 24 h post-infection (Figure 5C). This result shows the similar effect of the Pol fragment containing RT polymerase domain from three different isolates of subtype C virus on the reverse transcription, in spite of individual polymorphism of the AA sequences of RT (Figure 1) and different dynamics in disease progression in patients infected with these viruses. Our findings suggest that observed differences in reverse transcription efficiency are dependent on the viral subtype.

**Figure 6:**
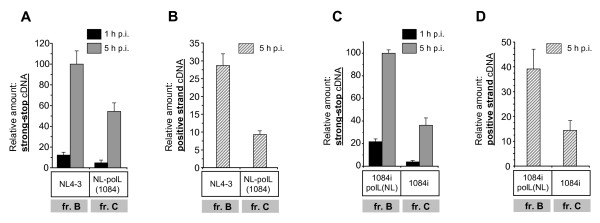
**The presence of RT polymerase domain from HIV-1 subtype C leads to decreased cDNA accumulation in reverse transcription complexes**. Accumulation of strong-stop (A and C) and positive-strand (B and D) viral DNA in RTCs isolated at 1 and 5 h p.i. Sup-T1 cells were synchronously infected with MLV Env-pseudotyped backbone NL4-3 or chimeric NLpolL(1084) (A and B), and backbone HIV1084i or chimeric 1084polL(NL) viruses (C and D). RTCs were purified from cell lysates. DNA was isolated from RTCs and subjected to quantitative real-time PCR. Levels of cDNA are shown as percentages of the maximal accumulation detected for strong-stop cDNA in RTCs. Error bars show the standard deviation from three independent viral preparations.

Since RTCs are undergoing proteasome-mediated degradation in the cytoplasm and two thirds of them have been shown to be degraded by several hours post-infection [42], the ratio of the reverse transcription products in cells infected with different virus strains shown in previous experiments, could be affected by intracytoplasmic degradation of RTCs. To minimize the effect of host cell-mediated degradation of RTCs on reverse transcription, we quantitatively analyzed the cDNA in RTCs isolated from the cytoplasm during the first five hours after infection with subtype B NL4-3, subtype C 1084i, or with chimeric viruses NL-polL(1084) and 1084-polL(NL). Since NL and 1084 viral vectors have different tropism, all viruses were pseudotyped with Env glycoprotein of the amphotropic murine leukemia virus (MLV). To ensure similar levels of viruses have entered regardless of the virus backbone and source of the inserted fragment, we measured p24^CA ^content in the RTCs isolated at 1 h after infection, since capsid protein was shown to remain associated with the viral core for hours after infection until completion of the reverse transcription [43,44]. We found that the p24^CA ^level was similar in early RTCs within virus strains of the same backbone. Differences in p24^CA ^levels between control backbone and chimeric viruses did not exceed 20% (data are not shown). However, analysis of the accumulation of reverse transcription products in the RTCs revealed significant differences between viruses containing the protease and RT polymerase domains from the NL4-3 and 1084i isolates regardless of the backbone vector (Figure 6). The RTCs of viruses carrying the subtype B RT polymerase domain, harvested at 1 h post-infection displayed a 2.5- (NL backbone) and 5-fold (1084i backbone) higher relative amount of strong-stop cDNA with respect to those carrying the 1084i RT polymerase domain (Figure 6A and 6C). The ratios of early cDNA between these strains, measured at 5 h after infection, were about 2x for NL backbone and 2.5x for 1084i backbone viruses. Similar results were observed in accumulation of the positive-strand DNA (Figures 6B and 6D) measured at 5 h post-infection, suggesting that the difference in cDNA accumulation between the viruses with RTs from B and C subtypes are dependent on the initial steps of the reverse transcription.

Taken together our data indicate that the presence of the RT, as well as only the polymerase, or the connection and RNase H domains of RT from subtype C viruses leads to a lower level of accumulation of strong-stop cDNA and late reverse transcription products, in both intact virions and intracytoplasmic RTCs independent of the virus backbone. The difference in viral DNA accumulation between viruses carrying RT from subtype B and C isolates may eventually determine the overall level of viral replication, that is consistent with the published data on subtype-associated effect of RT on viral replicative fitness [29].

### Cells infected with viruses carrying RT functional domains from HIV-1 subtype C isolates display decreased viral DNA integration

**Figure 7:**
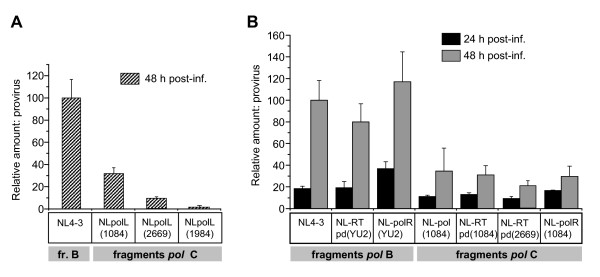
**Presence of RT functional domains from HIV-1 subtype C isolates correlates with decreased level of viral DNA integration**. **A **- Integration of cDNA of NL4-3 or NL-based viruses carrying protease and RT polymerase domains from subtype C isolates in Sup-T1 cell DNA at 24 h p.i. Cells were infected as described in the legend to Figure 5B. Total DNA was harvested and relative amounts of proviral DNA were measured using two-step *Alu*-based nested PCR assays as described in Materials and methods. Levels of provirus are shown as percentages of the maximum levels of integration detected in cells infected with NL4-3. Error bars show the standard deviation of three independent viral preparations. **B **-Integration of the backbone NL4-3 and chimeric viruses in Sup-T1 cells at 24 and 48 h p.i. DNA from the infected cells was harvested and subjected to quantitative real-time PCR as described in A. Levels of proviral DNA are shown as percentage of those detected in cells infected with NL4-3 at 48 h p.i. Results are mean ± SD of three independent experiments.

Lower levels of accumulation of reverse transcription products in viruses carrying subtype C *pol *products may correlate with the level of viral DNA integration into the host chromosomes. We then analyzed integration of these viruses using a two-step *Alu*-based nested PCR assay [45,46]. Quantitative analysis of the cellular DNA showed that viruses carrying protease and RT polymerase domains from different subtype C isolates, NLpolL(1084), NLpolL(2669) and NLpolL(1984), displayed between three- to fifty-fold fewer proviruses than subtype B NL4-3 (Figure 7A). To further confirm that this difference is due to the functional domains of RT, we compared various recombinant viruses that carry only the polymerase domain from subtype B [NL-RTpd (YU2)] or subtype C [NL-RTpd (1084) and NL-RTpd (2669)] isolates with virus strains carrying the whole Pol fragment without protease, or the connection, RNase H, and the integrase sequences from subtype B and C isolates. As expected, subtype B NL-RTpd (YU2) had similar levels of integrated provirus as NL4-3 (Figure 7B, left two pairs of columns). The two viruses carrying subtype C RT polymerase domain had 2-2.5-fold lower levels of integration at 24 h and 3- and 4-fold lower at 48 h post-infection (Figure 7B, 5^th ^and 6^th ^pairs of columns vs 1^st ^and 2^nd^). These findings are consistent with our data on cDNA accumulation in the virions and RTCs. Our results also showed that the integrase from B and C subtypes did not significantly affect the integration rate of the viruses containing B and C RT domains (Figure 7B, 2^st ^and 3^rd ^sets of bars vs. 5^th ^and 7^th^). Analysis performed at 48 h post-infection showed a mean of threefold higher levels of integration than at 24 h post-infection. Taken together, our data suggest that differences in the kinetics of cDNA accumulation in the RTCs are reflected in the levels of viral DNA integration.

### Viruses carrying RT polymerase domain from isolates of B and C subtypes do not show differences in the mutational rate

Differences in cDNA accumulation between viral stains carrying *pol *gene fragments from B and C subtypes are likely to be dependent on the *in vivo *RT enzymatic activity. To test whether these differences correlate with the fidelity of reverse transcription, we analyzed the frequencies of point mutations in the RT sequences of wild-type NL4-3 and chimeric NL-polL(1084) viruses after 27 days of infection in H9 cells. We analyzed a total of 28 individual sequences of the 750 base RT encoding fragment (codons 16-266) from NL4-3 and 43 sequences from NL-polL(1084) provirus using single viral genome PCR and sequence analysis [47]. Changes were observed when compared to the initial viral sequences. However, comparison of the RT encoding fragment sequences with the parental isolates did not show a significant difference in the frequencies of the nucleotide substitutions in this region of *pol *between NL4-3 and NL-polL(1084) viruses (Table 1, column 2).

**Table 1:** The substitution rate, frequency of G-to-A and silent mutations, and the ratios of non-synonymous to synonymous mutations in RT-encoding fragment (codons 25-250) of NL4-3 and NLpolL(1084) viruses.

	**Substitution rate**^**a**^			
		
Viral Strain	Total mutations **× 10**^**-4 **^**per nucl**.	G-to-A mutations **× 10**^**-4 **^**per nucl**.	Silent mutations **× 10**^**-4 **^**per nucl**.	**dN/dS**^**b**^
NL4-3	6.39 ± 1.88	2.35 ± 1.10	1.19 ± 0.82	4.28
NLpolL(1084)	7.83 ± 1.75	1.97 ± 0.76	1.64 ± 0.70	3.62

^a ^calculated as a mean frequency of mutations per nucleotide for each viral strain on the base of single genome PCR and sequence analysis of proviral genomes.

^b ^ratio of non-synonymous to synonymous mutations per site.

To test for the potential impact of deamination on mutation frequency in both virus strains, we separately determined the ratio of G-to-A substitutions, which may be a result of editing by APOBEC cytidine deaminases (reviewed in [48]). The detected G-to-A substitutions were located in the known positions which were described earlier for RT domain [49]. However, we did not detect significant differences in the frequency and proportion of G-to-A mutations between NL4-3 and NL-polL(1084), and both viruses demonstrated a similar G-to-A substitution rate of about 2 × 10^-4 ^(Table 1, column 3). Alignments of the RT encoding region revealed similar synonymous mutation rates for both virus strains of about 1.5 × 10^-4 ^(Table 1, column 4). However, the rate of non-synonymous substitutions (dN) was approximately fourfold higher than the rate of synonymous mutations (dS) (Table 1, column 5), indicating a high potential for positive selection for both viruses [50].

## Discussion

Genetic diversity of the *pol *gene among HIV-1 clades has been reported primarily in the context of drug resistance manifestation [5,15,27,30,51-53], and reviewed previously [2,54]. In this study, we have demonstrated a correlation between the presence of either the whole RT, or only the N-terminal (polymerase), or C-terminal (connection and RNase H) domains of RT from the HIV-1 subtype C and a decreased level of viral replication, cDNA accumulation in virions or cytoplasmic RTCs, and integration. The C-terminal Gag region (part of NC, sp1, and p6^Gag^), the protease, as well as the integrase and Vif protein of subtype C viruses did not seem to play a substantial role in lower levels of cDNA accumulation, integration, and the overall virus replication when compared to subtype B viruses.

Our data indicate that the RT polymerase domain from subtype C alone significantly affected the accumulation of negative strand strong-stop DNA and late DNA products, demonstrating the importance of this domain for subtype-specific differences in reverse transcription. However, the viruses with the chimeric RT, which contains the connection and RNase H domains from clade C and polymerase domain from clade B, also demonstrate decreased levels of accumulation of early cDNA products in permeabilized virions (Figure 5A), even though RNase H enzymatic activity is not required for the minus-strand strong-stop DNA synthesis [25]. Since the RNase H domain has been shown to profoundly affect the functions of the polymerase domain [55,56], our findings suggest that the C-terminal part of RT from subtype C viruses influences the polymerase domain of subtype B RT in the chimeric constructs. This effect results in a decreased efficiency of reverse transcription in the virions and RTCs of recombinant viruses. Therefore, the observed high level of cDNA accumulation in subtype B virus probably involves a cooperative effect of both the N- and C-terminal ends of the RT molecule, whereas the presence of the whole RT from subtype C virus, as well as chimeric B-C RT resulted in low level of cDNA accumulation (Figure 5 and 6).

Our results also showed that the efficiency of DNA integration for viruses carrying subtype C *pol *fragments is always lower than those with *pol *from subtype B isolates, even though the integrase gene were identical. This observation, together with published data demonstrating the similarity between the integrase of B and C subtypes [24], suggests that the differences in the level of integration may be an outcome of the differences in the accumulation of integration-competent reverse transcription products. The RT may still be playing a major role in contributing to the differences observed in early replication events and the overall level of replication between subtype B and C viruses. Moreover, we expect that the delayed reverse transcription, related viral uncoating or other pre-integration events of subtype C viruses may extend the presence of the RTCs in the cytoplasm. Since RTCs undergo proteasome-mediated degradation in the cytoplasm [42], an extended presence of subtype C RTCs in this compartment may increase the risk of their degradation in the proteasoms, thereby decreasing the level of viral DNA integration and overall viral replicative capacity.

Our analysis of the RT sequences of clade B and C viruses did not reveal any clade-specific AA differences in their functionally important regions. The AA motifs of the polymerase domain, responsible for polymerase activity, primer grip, proper dNTP positioning, and coordination of triphosphate moiety, as well as catalytically important residues in the RNase H domain are identical in all the studied isolates from both subtypes (Figure 1). However, the distinct subtype-specific AA changes in functionally non-important regions may indirectly affect the RT function. Quan and colleagues suggested that typical for subtype C viruses T39K/E and Q207E/R substitutions located in the middle of the αA and αF helices can potentially disturb structures in the finger subdomain of RT [28]. Our analysis of the potential effect of the detected AA changes in the RT polymerase domains of B and C subtypes on the secondary structure of the p66 subunit of RT, performed using Network Protein Sequence Analysis software [57], also indicated that there are some differences located in the regions between AA 128 to 168 and 214 to 246 (data not shown). Since these regions include functionally important LPQG (149-152) and LWMGYELH (228-235) motifs (Figure 1, grey boxes), and are located near the β strand of YMDD motif, we anticipate that subtype-specific AA differences may affect the net charge at their positions and hereby facilitate the conformational changes of the functionally important RT regions. We expect that our observed subtype-specific AA differences in the region of RT polymerase domain, surrounding catalytic Asp185 and Asp186 residues, as well as AA changes in the variable regions of the RNase H domain in clade C viruses may eventually influence the RT activity, resulting in slower kinetics of accumulation of the DNA products.

Earlier studies of the DNA polymerase activity and RT inhibitor susceptibilities of the recombinant RTs from different subtypes of HIV-1, performed using synthetic RNA or DNA substrates [28,58,59], did not reveal differences in basic RT activity between subtypes. However, since the RT kinetics and processivity have been shown to be dependent on the sequence of the RNA template [60,61] and affected by the viral NC protein, which is essential for proper tRNA binding [62], strand transfer [63,64], and RNase H activity modulation [65], the biochemical analysis of recombinant RT enzymes with synthetic substrates *in vitro *may not necessarily reflect their activities *in vivo *during virus infection. Identification of the molecular determinants of subtype-specific differences in RT function *in vivo *will be the focus of our future studies.

Taken together, our results show that RTs of B and C subtypes display functional difference in HIV-1 infection, suggesting that this difference is one of the important factors affecting replication capacity and lower cytopathogenicity of subtype C isolates. These data provide new insight into the functional diversity of HIV-1 subtypes. Our findings may also contribute to optimization of HIV-1 subtype-specific therapy, and would facilitate the development of new ART strategies.

## Materials and methods

### Plasmid Constructs

The HIV-1 proviral clones NL [33] and HIV1084i [66] were used as the source of reference viruses and vectors for cloning of the HIV-1 *pol *gene fragments (Figure 2A and 2B). To create the backbone subtype C vector for recombinant clones, complete 1084i provirus was excised from the parental pCR2.1 Topo cloning vector (Invitrogen, Carlsbad, CA) by NotI (all restriction enzymes were from New England Biolabs) and subcloned into the same vector, previously cleaved with NotI and PspOMI to provide compatible ends and to remove the 28 base fragment of the multicloning region. Fragments of the HIV-1 DNA genome, encoding 26 C-terminal amino acids (AA) of the nucleocapsid (p7 NC) and p6 protein of Gag, complete protease, and 312 (for subtype C) or 367 (for subtype B) N-terminal AAs of RT were amplified from NL and HIV1084i clones or from 1984i and 2669i proviral DNA of subtype C HIV-1 primary isolates. Primers B1339p7F (5'-AAATTGCAGGGCCCCTAGGAAAAAGGGCTGTTG-3'), containing a PspOMI restriction enzyme site, and 2992p51R (5'-GCCTCTGTTAACTGTTTTACATCATTAGTGTGG-3') with an introduced HpaI site, were used for PCR amplification of the NL4-3 DNA fragment. Forward primer C1339p7F (5'-AAATTGCAGGGCCCCCAGGAAAAAGGGCTGTTG-3'), also containing the PspOMI site, and reverse primer C3478p51R (5'-CCATGTACCGGTTCTTTTAAAATTTCCCTG-3') with an introduced AgeI site were used for PCR amplification of the homologous fragments from 1084i, 1984i and 2669i DNA. The fragments were first subcloned into the pGEM-T Easy vector (Promega, Madison, WI), and the inserts were then used to replace the homologous fragments in the HIV-1 proviral clones HIV1084i or NL4-3 (Figure 2F and 2G). For cloning of the *pol *gene fragment encoding the RT polymerase domain, the DNA sequence containing 124 nt from the protease encoding region and RT polymerase domain was amplified by PCR from subtype B YU-2 molecular clone with forward primer F-NLpr-BclI (5'-ACAGTATGATCAGATACTCATAGAAATCTGCGG-3') containing BclI restriction enzyme site and reverse primer polCR2 (5'-ATACTCCATGTACCGGTTCTTTTAGAA-3') with an introduced AgeI site. Identical fragments from subtype C molecular clone HIV1084i and primary provirus 2669i were PCR amplified with forward primer F-Cpr-BclI (5'-ACAGTATGATCAGATACTTATAGAAATTTGTGG-3'), which also contains the BclI site, and polCR2 primer. The fragments were then subcloned into the pGEM-T Easy vector and transformed into *dam*^-^/*dcm*^- ^Competent *E. coli *(New England BioLabs) since BclI is susceptible to the *dam *methylation. The DNA fragments after digestion with respective restriction enzymes were then ligated with the linearized HIV-1 NL4-3 proviral clones to replace the host gene fragments (Figure 2D). To clone the DNA fragments encoding the RT connection and RNase H domains, the integrase, and the Vif into the NL vector, the fragments were PCR amplified from YU-2 and HIV1084i proviral clones with forward primer RTage1F (5'-TAAAAGAACCGGTACATGGAGT-3') with an introduced AgeI site and reverse primer polEcoR1R (5'-TTGTTGCAGAATTCTTATTAT-3') containing the EcoRI restriction enzyme site. After subcloning into the pGEM-T Easy vector, the fragments were ligated either into NL4-3 proviral clone (Figure 2E), or into the recombinat NL-based vectors containing the RT polymerase domain, encoding the *pol *gene segment from 1084i isolate, to generate the chimeric subtype B virus carrying the entire RT from subtype C isolate (Figure 2C).

### Cells and Viruses

293T/17 and H9 cells were purchased from ATCC (Manassas, VA). Sup-T1 (from James Hoxie), MAGI (from Michael Emerman), and TZM-bl (from John Kappes and Xiaoyun Wu) were provided by the NIH AIDS Research & Reference Reagent Program. U87.CD4.CCR5 cells were kindly provided by Lee Ratner from Washington University. All cell cultures were maintained under conditions recommended by the providers.

HIV-1 backbone and recombinant virus stocks were prepared by transfecting 293T/17 cells with provirus-encoding plasmids using Metafectene (Biontex, Planegg, Germany). The DMEM media was replaced with RPMI-1640 about 18 h after transfection. At about 30 h the supernatants were harvested and filtered through a 0.45-μm filter. The 50% tissue culture infective dose of each virus stock was determined by single infection cycle assay using 10^5 ^HeLa-CD4-LTR/β-gal (MAGI) indicator cells [67] for the NL4-3 backbone viruses, or TZM-bl cells for the 1084i backbone viruses, with fourfold serial dilutions of viruses as described previously [68].

To generate HIV-1 viruses for analysis of reverse transcription, nuclear import, and integration, 293T/17 cells were transfected with different HIV-1 proviral clones alone or with the pcDNA-Env(MLV) plasmid (kindly provided by Nathaniel Landau) at a 4:1 ratio using Metafectene as described earlier (23). The resulting viruses were then incubated for 2 h at 37°C in a buffer containing 10 mM MgCl_2 _and 50 U/ml of RNase-free DNase I (Roche, Indianapolis, IN). Virus particles were further concentrated by centrifugation through a 30% sucrose cushion in PBS at 24,000 RPM in a Beckman SW-28 rotor for 2 h at 4°C. Virus pellets were resuspended either in RPMI medium containing 20 mM HEPES pH 7.4 (for infection) or in PBS for RNA isolation and Western blot analysis. For infection viral titers were normalized to 0.01 or 0.1 pg of p24^CA ^per cell, using a p24 ELISA kit (PerkinElmer, Waltham, MA). Infection of Sup-T1 cells was performed in 12-well plates (3 × 10^6 ^cells per well) by spinoculation at 1000 × g for 2 h at 18°C, according to the published protocol [69]. The cells were washed twice with PBS at room temperature and incubated in culture medium at 37°C for 0-48 h. For infection with nevirapine, the cells were pre-treated overnight with 10 μM nevirapine (AIDS Research and Reference Reagent Program) and then cultivated for 24 h after infection in the fresh culture media containing 10 μM nevirapine.

### Western blot analysis

The suspensions of virus particles in PBS were mixed with equal volumes of Laemmli Sample Buffer (BioRad), heated in boiling water for 2 min and then subjected to SDS-PAGE. Proteins were transferred to PVDF membranes, and detected using anti-HIV-1 p24 (24-3) or anti-HIV-1 integrase (2C11) mouse monoclonal antibodies from NIH AIDS Research & Reference Reagent Program, or anti-HIV-1 RT (ab9066) monoclonal antibody from Abcam. The HIV-1 GagPol polyprotein was identified using human HIV immunoglobulin (HIV-IG) also from NIH AIDS Research & Reference Reagent Program. Specific bands were visualized by ECL (Thermo Scientific, Rockford, IL). Quantification of the Western blotting results was performed using ImageJ software.

### Endogenous reverse transcription (ERT) in viral particles

Preparations of viral particles containing 100 ng of p24^CA ^were used for ERT assay. The virus particles were incubated with or without (control) dNTP mixture (1 mM) for 1.5, 2, 3, and 5 h at 37°C in ERT buffer (5 mM MgCl_2_, 1 mM DTT, and 15 μg/ml melittin in PBS) as previously described [40,41]. Samples were collected and DNA was purified with 25 μg of glycogen using IsoQuick DNA Extraction Kit (ISC BioExpress, Kaysville, UT). RT products were analyzed by real-time PCR with primer sets specific for strong-stop viral DNA as described below.

### RNA purification and RT reaction

RNA was purified from suspensions of virus particles containing 250 ng of p24^CA ^using RNA STAT-50LS RNA isolation solution (Tel-Test, Friendswood, TX) according to manufacturer's protocol. Reverse transcription of isolated RNA to cDNA for subsequent quantitative real-time PCR analysis was performed using GeneAmp RNA PCR Kit components (Applied Biosystems, Foster City, CA) and the oligo-dT primer according to manufacturer's protocol.

### Reverse transcription complex (RTC) isolation and purification of DNA from RTCs and cell lysates

Approximately 5 × 10^6 ^infected Sup-T1 cells were collected and washed twice with 40 ml cold PBS. For quantitative analysis of cDNA and proviral DNA, total DNA was purified using the IsoQuick DNA Isolation kit and then analyzed by real-time quantitative PCR. Fractionation of cells and isolation of the RTCs was performed according to Fassati and Goff [70] with modifications as described previously [71]. Briefly, harvested cells were washed with cold PBS and homogenized in cold hypotonic buffer supplemented with 0.025% Brij 96 using EZ-Grind kit (G Biosciences, St. Louis, MO). Viral RTCs were purified from total cell homogenates by centrifugation through a 50% sucrose cushion in hypotonic buffer at 100,000 × g in a Beckman MLS-50 rotor for 3 h at 4°C. Pelleted HIV-1 RTCs were resuspended in 200 μl of buffer K [20 mM HEPES, pH 7.3, 150 mM KCl, 5 mM MgCl_2_, 1 mM dithiothreitol, and 0.01 volume of Halt protease inhibitor cocktail (Pierce, Rockford, IL)] and stored at -80°C [72]. DNA from RTC suspensions containing about 500 pg p24^CA ^(as detected by p24^CA ^ELISA) was extracted using the IsoQuick DNA Isolation kit with an addition of 25 μg of glycogen (Invitrogen, Carlsbad, CA) in each RTC sample.

### Quantitative PCR

DNA from purified viral RTCs was analyzed by real-time PCR using two sets of primers. The first set detects the negative-strand "strong-stop" DNA (the early reverse transcription product) and consists of forward primer M667 (5'-GGCTAACTAGGGAACCCACTG-3'), reverse primer AA55 (5'-CTGCTAGAGATTTTCCACACTGAC-3'), and probe Er-LTR (5'-FAM-GTCACACAACAGACGGGCACACACTA-TAMRA-3') specific for the R-U5 region of the HIV-1 LTR. The second set recognizes the positive-strand DNA (late reverse transcription product) and consists of primers: FOR-LATE (5'-TGTGTGCCCGTCTGTTGTGT-3'), REV-LATE (5'-GAGTCCTGCGTCGAGAGATC-3'), and probe Lt-LTR-Prb (5'-FAM-CAGTGGCGCCCGAACAGGGA-TAMRA-3') specific for the U5-Ψ LTR region [45]. PCR reactions were performed with PerfeCTa qPCR FastMix, UNG (Quanta Biosciences, Gaithersburg, MD) using 300 nM of each primer and 200 nM probe. The conditions used were: one cycle at 45°C for 2 min, and at 95°C for 4 min, then 15 sec at 95°C, and 30 sec at 60°C for 45 cycles. Serial dilutions of DNA from 8E5 cells (CEM cell line containing a single copy of HIV-1 LAV provirus per cell) were used as the quantitative standards [73]. Quantitative analysis of 2-LTR circles was performed according to published protocol [45]. The 2-LTR standard was kindly provided by Michael Bukrinsky. The Real-time PCR assay was performed with forward primer MH535 (5'-AACTAGGGAACCCACTGCTTAAG-3'), reverse primer MH536 (5'-TCCACAGATCAAGGATATCTTGTC-3'), and probe MH603 (5'-FAM-ACACTACTTGAAGCACTCAAGGCAAGCTTT-TAMRA-3'). Viral 2-LTR circles were detected from 500 ng total cellular DNA with PerfeCTa qPCR FastMix, UNG. Reaction conditions were the same as described above. Two-step nested PCR assays were used for quantitative HIV-1 DNA integration analysis. The first round PCR was performed in a 25 μl reaction mix as described previously [46]. Briefly, 100 nM of the genomic *Alu *forward primer, Alu-F (5'-GCCTCAATAAAGCTTGCCTTGA-3'), 600 nM of HIV-1 *gag *reverse primer, Gag-R (5'-GCTCTCGCACCCATCTCTCTCC-3'), and 100 ng of cellular genomic DNA were mixed with 1.5 mM MgCl_2_, 0.25 mM dNTPs, 0.05 U of Platinum *Taq *DNA polymerase (Invitrogen) and *Taq *polymerase reaction buffer (Invitrogen). The conditions were 2 min hot start at 94°C, then 30 sec at 93°C, 1 min at 50°C, and 2 min at 70°C for 20 cycles. The second round was performed with 5 μl of the material from the first round in 20 μl of reaction mix. The primer set and reaction conditions were the same as for quantitative detection of the positive-strand HIV-1 DNA described above. Serial dilutions of DNA from 8E5 cells were used to calculate the relative copy numbers of integrated DNA. To normalize integration data relative to target cell DNA, a quantitative real-time PCR of β-globin DNA was performed using the forward primer BGF1 (5'-CAACCTCAAACAGACACCATGG-3'), reverse primer BGR1 (5'-TCCACGTTCACCTTGCCC-3'), and probe BGX1 (5'-FAM-CTCCTGAGGAGAAGTCTGCCGTTACTGCC-TAMRA-3'). Real-time PCR reactions were carried out at least in triplicate using the iCycler with iQ Multicolor Real-time PCR Detection System (BioRad) and iCycler software.

### *In vitro *virus replication

Experiments were performed using procedures described previously [74]. Sup-T1 cells (1 × 10^6 ^in 1 ml of culture medium) were exposed to backbone NL4-3 (X4 isolate) or recombinant NL-based viruses normalized to 0.01 pg of p24^CA ^per cell for 4 h. The cultures were subsequently maintained in 1 ml of growth medium. Every 3 to 4 days, 100 μl of cultures were placed into 900 μl of medium containing 0.9 × 10^6 ^uninfected Sup-T1 cells. The remaining culture medium and cell pellets were collected. Virus replication was monitored by syncytium formation and then quantitated using p24^CA ^ELISA of the culture supernatants. Harvested cells were used for isolation of total cellular DNA. Cell viability was measured using Vi-Cell cell viability analyzer (Beckman Coulter). Cellular DNA was purified with IsoQuick DNA Isolation kit for sequence analysis.

Similar experimental procedures were performed to analyze infection by HIV1084i and chimeric 1084polB(NL) viruses in U87.CD4.CCR5 cells. Two hundred fifty thousand cells were infected by spinoculation with 2.5 ml of virus suspension per well (MOI = 0.05) in a 6-well tissue culture plate. The cells were lifted mechanically every 3-4 days using cell lifters (Corning, Lowell, MA) and resuspended in the culture medium by pipetting. Two hundred microliters of the suspension with 0.25 × 10^6 ^cells were incubated in 1.8 ml of fresh growth medium for subsequent cultivation. Virus replication was monitored by p24^CA ^measurement.

### Single-Genome Amplification and DNA Sequencing

SGA of the 750 base RT encoding fragment (codons 16-266) was performed from individual provirus sequence according to the described methods [47]. Samples of cellular genomic DNA were harvested from cultured cells on 27^th ^day of infection with NL and NLpolC(1084) virus strains The samples containing from 500 to 500 000 copies/μl of HIV-1 DNA (according to real-time PCR measurement) were diluted until approximately 30% of the PCR reactions yielded DNA product. The RT region of the provirus was amplified from diluted cellular DNA samples by nested PCR and used for sequence analysis. The first PCR round was performed with primers B1339p7F and 2992p51R. First-round PCR products (1 μl) were used for second round PCR for 25 cycles at 56°C annealing temperature with primers 881MF (5'-TGT AAA ACG ACG GCC AGT CCC GGG ATG GAT GGC CCA AAA GTT AAA CAA-3') and 891MR (5'-CAG GAA ACA GCT ATG ACC GCT AGC CCA ATT CAA TTT TCC CAC TAA-3'), containing the 17 nt M13 sequence at 5'-ends [75,76]. Second-round PCR products were purified with Perfectprep PCR Cleanup 96 kit (Eppendorf, Hamburg, Germany) and sequenced directly using both M13 forward (5'-GTAAAACGACGGCCAGT-3') and M13 reverse (5'-CAGGAAACAGCTATGAC-3') primers in BigDye Terminator v3.1 Cycle Sequencing master mix (Applied Biosystems, Foster City, CA). Sequences were analyzed with the 3100-Avant automated DNA sequencer (Applied Biosystems/Hitachi). Sequence data were manually edited with Sequencher, version 4.6, and CodonCode Aligner software (Gene Codes Corporation). From 25 to 43 individual sequences were obtained from each sample. Frequency of polymorphisms was calculated as a mean of the number of mutations per nucleotide for each viral genome. Analysis of synonymous versus nonsynonymous mutations relative to the initial HIV-1 RT reference sequences was performed using Highlighter software tool http://www.hiv.lanl.gov/content/sequence/HIGHLIGHT/highlighter.html.

## Competing interests

The authors declare that they have no competing interests.

## Authors' contributions

SI designed and carried out most of the experiments, analyzed data and prepared the manuscript; SI and MW performed the experiments on virus replication, isolation of RTCs and DNA sequence reactions; YF assisted in the preparation of recombinant viruses and Western blot analysis; CW oversaw the entire project and preparation of the manuscript. All authors read and approved the final manuscript.
